# Correlation between afferent rearrangements and behavioral deficits after local excitotoxic insult in the mammalian vestibule: a rat model of vertigo symptoms

**DOI:** 10.1242/dmm.024521

**Published:** 2016-10-01

**Authors:** Sophie Gaboyard-Niay, Cécile Travo, Aurélie Saleur, Audrey Broussy, Aurore Brugeaud, Christian Chabbert

**Affiliations:** 1 INSERM U1051, Montpellier 34090, France; 2 Sensorion, Montpellier 34000, France; 3 Aix Marseille University UMR 7260, 13331 Marseille, France

**Keywords:** Excitotoxicity, Calyx terminals, Hair cells, Physiopathology, Vestibule, Functional recovery

## Abstract

Damage to inner ear afferent terminals is believed to result in many auditory and vestibular dysfunctions. The sequence of afferent injuries and repair, as well as their correlation with vertigo symptoms, remains poorly documented. In particular, information on the changes that take place at the primary vestibular endings during the first hours following a selective insult is lacking. In the present study, we combined histological analysis with behavioral assessments of vestibular function in a rat model of unilateral vestibular excitotoxic insult. Excitotoxicity resulted in an immediate but transient alteration of the balance function that was resolved within a week. Concomitantly, vestibular primary afferents underwent a sequence of structural changes followed by spontaneous repair. Within the first two hours after the insult, a first phase of pronounced vestibular dysfunction coincided with extensive swelling of afferent terminals. In the next 24 h, a second phase of significant but incomplete reduction of the vestibular dysfunction was accompanied by a resorption of swollen terminals and fiber retraction. Eventually, within 1 week, a third phase of complete balance restoration occurred. The slow and progressive withdrawal of the balance dysfunction correlated with full reconstitution of nerve terminals. Competitive re-innervation by afferent and efferent terminals that mimicked developmental synaptogenesis resulted in full re-afferentation of the sensory epithelia. By deciphering the sequence of structural alterations that occur in the vestibule during selective excitotoxic impairment, this study offers new understanding of how a vestibular insult develops in the vestibule and how it governs the heterogeneity of vertigo symptoms.

## Abstract (precis)

**Summary:** Early sequence of afferent injury and repair in vestibular sensory epithelium that correlates with balance disorders and functional restoration is detailed in a rodent model of excitotoxicity.

## INTRODUCTION

Vestibular disorder symptoms include vertigo, dizziness, postural imbalance and lack of gaze fixation during head movements. Although they might have a central origin, it is commonly believed that they predominantly result from transient or permanent impairments of vestibular function in the inner ear (Brandt et al., 2009; Chabbert, 2013; Soto and Vega, 2010). The static deficits commonly encountered following a unilateral vestibular insult, consisting of ocular nystagmus and postural instability, are generally attributed to a functional imbalance between bilateral vestibular nuclei (Curthoys and Halmagyi, 1995; Dieringer, 1995). However, the mechanisms that support the vestibular dysfunction or lack of function have never been fully elucidated so far. This lack of knowledge impairs the development of pharmacological approaches aiming to efficiently protect the vestibule under pathological conditions.

Different aetiologies – including alterations of endolymph properties, insult to sensory cells or death, and uncoupling of vestibular afferents resulting from diverse pathogenic situations – have been proposed to explain sudden vestibular dysfunction. Among these pathogenic conditions – including ototoxicity, infections, ischemia, trauma, inflammation or aging – the impact of the excitotoxicity remains unclear. Often associated with ischemic conditions, clinical confirmation generally relies on identification of stroke or thrombosis with magnetic resonance imaging (MRI) (Iadecola and Anrather, 2011). However, excitotoxic damage can be far more subtle, remaining imperceptible with MRI. Excitotoxicity is primarily a pathophysiological phenomenon that affects specifically glutamatergic synapses (Olney, 1969). This process results from the excessive release of glutamate in the vicinity of presynaptic elements and its spill over within the synaptic cleft, and from the sustained activation of glutamate receptors located at postsynaptic terminals. Under these conditions, the prolonged afflux of cations in the postsynaptic terminal is accompanied by an influx of water that in turn induces swelling of dendrites and could lead to complete synaptic uncoupling.

The sensory structures of the inner ear, including hair cells and primary neurons, are excitable cells. Along with neurons from the central nervous system, they are particularly vulnerable to excitotoxicity. When exposed to ischemic and reperfusion conditions (Puel et al., 1995; Tabuchi et al., 2002), or acoustic trauma (Kujawa and Liberman, 2009; Puel et al., 1998), the cochlea exhibits characteristic patterns of excitotoxic damage. Synaptic damage and its associated functional impairments are significantly reduced in the presence of selective blockers of glutamate receptors (Puel et al., 1994, 1998). Based on these pre-clinical proofs of concept, clinical studies using similar types of protective approaches for auditory synapses have demonstrated a significant reduction in the tinnitus associated with acoustic trauma (van de Heyning et al., 2014). At the vestibular level, partial or complete degeneration of vestibular end organs has been demonstrated in humans in conditions of vertebrobasilar artery occlusion (Kitamura and Berreby, 1983; Oas and Baloh, 1992). This damage has been implicated in the acute vestibular disorders experienced by individuals. Excitotoxic insult of vestibular primary afferents has been proposed as a possible cause of vestibular neuritis (Dyhrfjeld-Johnsen et al., 2013). More recently, the notion of repeated excitotoxic insults in the vestibular sensory epithelia, as the result of blood perfusion alteration in the inner ear, has also been proposed to explain the iterative and progressive symptomology of Menière's syndrome (Foster and Breeze, 2013).

The recent development of animal and *in vitro* models of excitotoxically-induced vestibular insults has provided the opportunity to confirm the central role of glutamate in the excitotoxic cascade and to identify the cellular effectors involved (Brugeaud et al., 2007; Liu, 1999; Shimogori and Yamashita, 2004). Interestingly, some studies have reported the capacity of the neuronal vestibular network to spontaneously undergo repair following selective destruction of the vestibular primary synapses (Travo et al., 2012) and to support functional restoration (Brugeaud et al., 2007). Taken together, these studies highlight the cellular events that occur in the first days after the induction of the vestibular insult and that support excitotoxically-induced vestibular dysfunction. What is primarily lacking today is acquisition of information on what happens in the first hours after the onset of the insult. This information is essential for the development of early protection strategies to counteract the excitotoxically-induced vestibular insult.

The present study was designed to directly address this gap in our understanding. Using an excitotoxic paradigm to mimic vestibular pathological conditions, we detailed the time course of induced balance disorders using behavior testing, together with cellular changes that occur at the contact between vestibular hair cells and their nerve afferent terminals by using immunohistochemistry, as well as light, electron and confocal microscopy. We report a direct correlation between the peak crisis of vestibular disorder symptoms and severe damage to afferent terminals in the very early stages of the response to insult. This study also confirms the remarkable plasticity of the vestibular afferent terminals to engage a repair process following excitotoxic injury.

## RESULTS

### Altered vestibular behavior following transtympanic injection of kainic acid

**Fig. 1.**
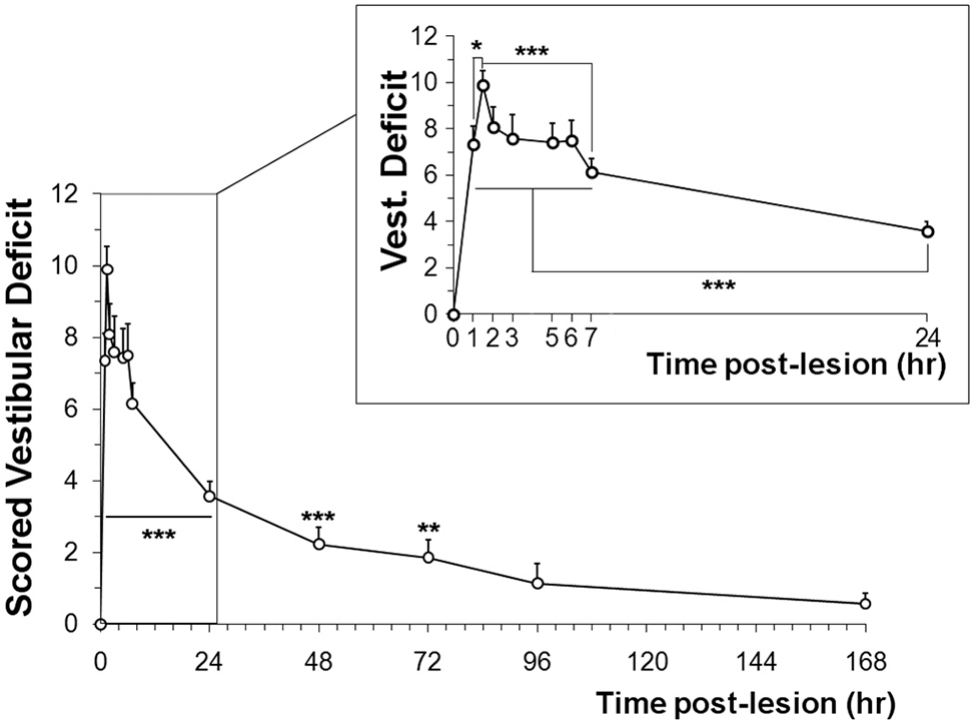
**Vestibular dysfunction induced by a single transtympanic injection of kainic acid.** As soon as 1 h after the injection, vestibular dysfunction was induced. The peak crisis was maximal at 1.5 h and lasted until 7 h post injection (*P<0.05 vs 1 h, ***P≤0.001 vs 7 h). Then, the cumulative score decreased and was no longer statistically different from control animals at 96 h. Significance of the ANOVA followed by the Dunn's method to analyze the whole time post-lesion, ***P≤0.001, **P≤0,01. Mann–Witney test was used to compare with control animals that had been injected with the saline solution (*n*=8). Error bars are s.e.m.

Single and unilateral (right ear) transtympanic injection of kainic acid [single transtympanic kainic acid (STTK) injection, 12.5 mM, 100 µl] rapidly elicited stereotypical vestibular dysfunction that was characterized by circling behavior, walking backward and head bobbing when exploring. The rat also displayed body torsion during tail hanging and failed to rectify full supine position in the air righting or contact inhibition reflex tests. Vestibular dysfunction scores were collected according to the method previously described (Brugeaud et al., 2007; Desmadryl et al., 2012; Llorens et al., 1993; Llorens and Rodriguez-Farre, 1997). The time course of the onset and progress of the vestibular dysfunction was studied at different time points from 1 h to 1 week following the STTK injection (Fig. 1).

Following the STTK injection, pronounced altered vestibular behavior was evident as soon as the rats awakened from anesthesia. Altered vestibular behavior developed in three distinct phases. An initial phase was identifiable within the first two hours following the insult induction (Fig. 1, insert). One hour following the insult induction, the mean vestibular dysfunction score had already exceeded 7 (7.34±0.77, *n*=29; mean±s.e.m.), and peaked at 9.90±0.62 (*n*=20) 1.5 h after administration of the kainate. The mean peak score statistically differed from the score at 1 h after the induction (*P*=0.018). A second phase with quite stable though highly pronounced vestibular dysfunction lasted between 2 and 7 h [8.08±0.84 (*n*=23) and 6.15±0.58 (*n*=26) at 2 and 7 h, respectively] after the initial insult. Subsequently, a third phase of progressive recovery occurred with slowly reduced vestibular dysfunction. At 96 h post induction, this phase ended with a complete withdrawal of vestibular dysfunction. At this time, mean score values of 1.14±0.55 (*n*=7) did not statistically differ from those obtained in normal pre-lesioned animals (*P*=0.125).

In summary, following unilateral excitotoxic insult, three different phases of altered behavior were observed: (1) a fast and acute crisis within 2 h following the insult, (2) a stable phase of pronounced vestibular dysfunction lasting 7 h after the induction and (3) a slower recovery period that lasted 4 days before normal functional behavior was restored.

### Evidence of excitotoxic damage at vestibular afferent terminals in the first hours following transtympanic injection of kainic acid

**Fig. 2.**
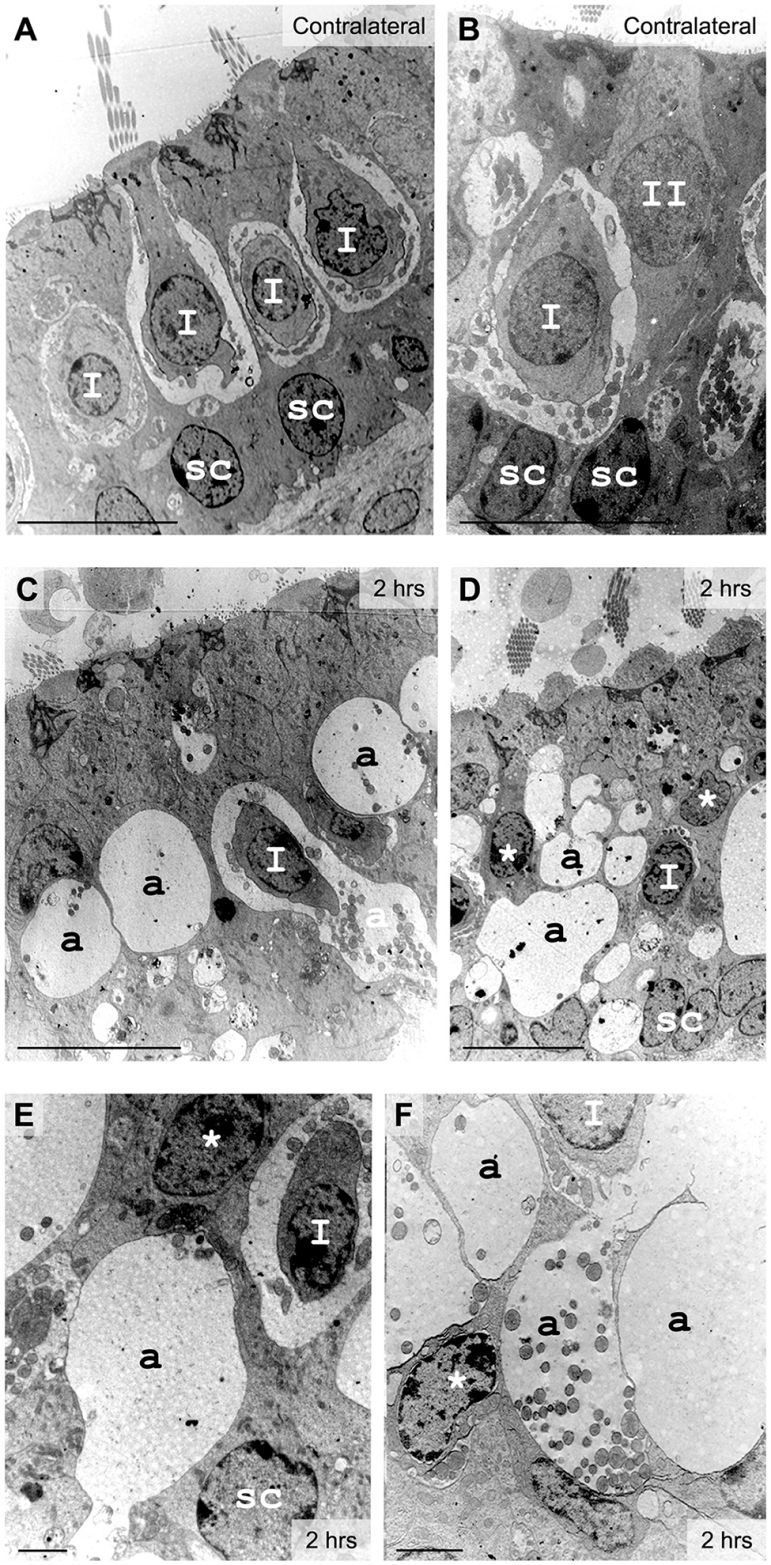
**Excitotoxic injury in vestibular utricles observed 2 h after lesion formation with electron microscopy.** (A,B) In control tissue, type I hair cells (I) surrounded by calyx nerve endings and cylindrical type II hair cells (II) were identified lying over the nuclear layer of supporting cells (sc). (C-F) In injured inner ear, a few type I hair cells could still be identified from the calyx terminal. Most hair cell types became undetermined (*) because of their distorted morphology induced by large swellings at their base (a). Localization and mitochondrial content was used to characterize the swollen structures as afferent terminals (a). Scale bars: 10 µm (A-D); 2 µm (E,F).

**Fig. 3.**
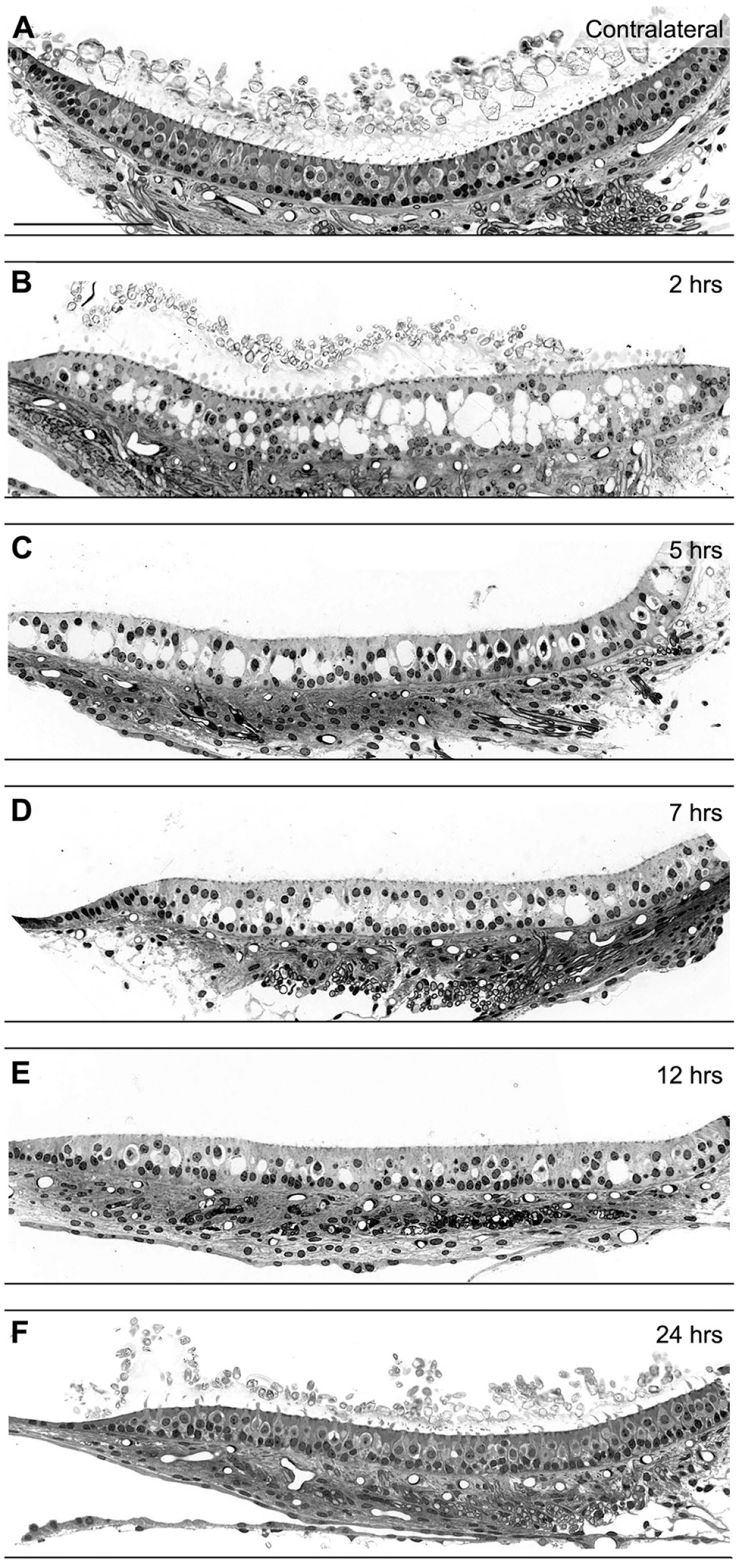
**Changes over time in the general organization of vestibular epithelia after induced excitotoxic injury.** The gross morphology was observed on semi-thin sections of vestibular utricles. (A) In control ears, the regular macro-organization of hair cells and supporting cells constitutes the sensory epithelium lying on a conjunctive tissue, where innervating fibers arrive. (B) 2 h after the STTK injection, large holes appeared between the layers of nuclei of hair and supporting cells. (C-E) Between 7 h and 12 h post lesion, the size of swellings progressively decreased. (F) At 24 h post lesion formation, no more vacuoles were observed in the sensory epithelium, whose gross morphology appeared similar to the contralateral one. Notice the innervating fibers were always present underneath the injured sensory epithelium. Scale bars: 100 µm.

**Fig. 4.**
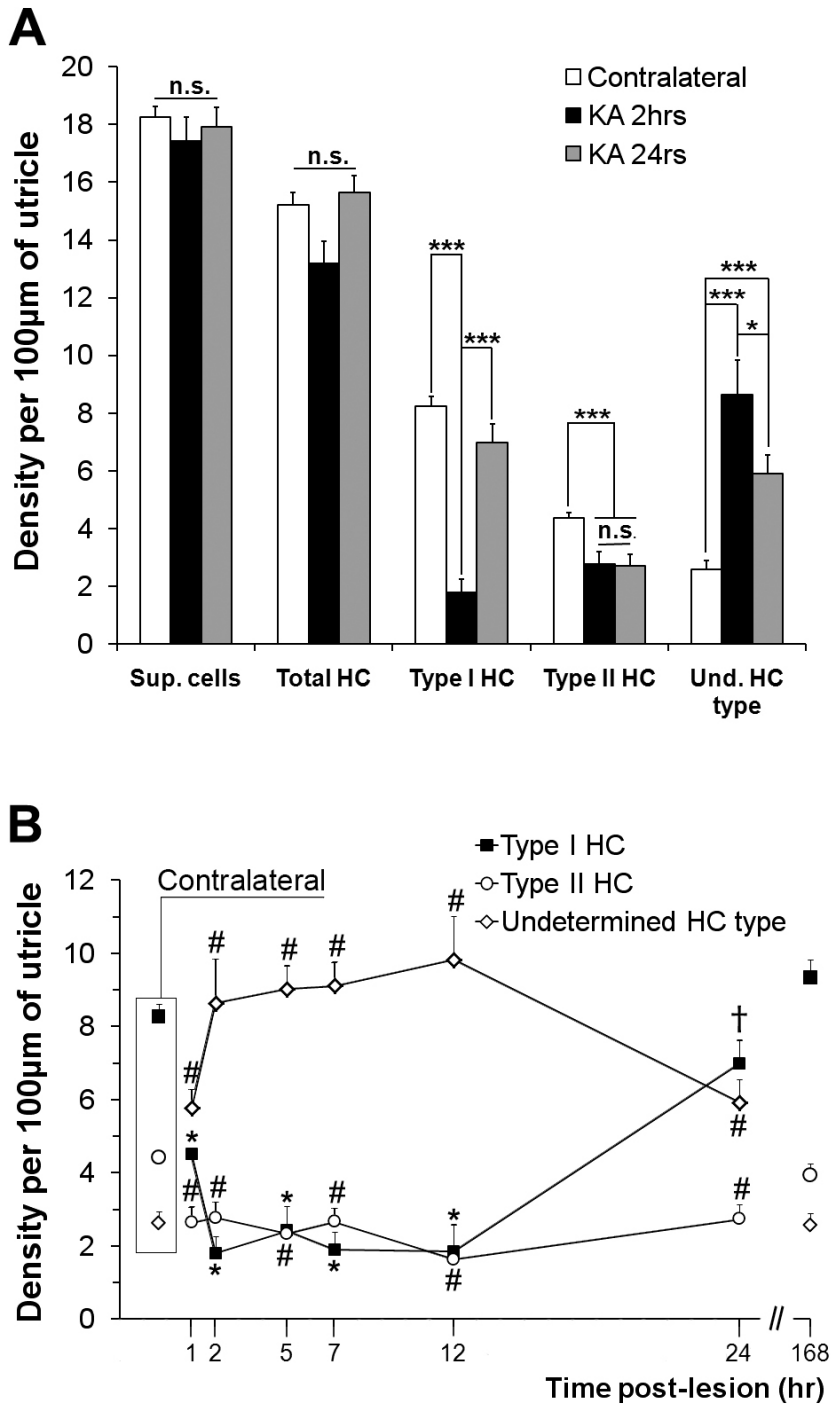
**Density of hair cells and supporting cells in vestibular epithelia following the excitotoxic injury.** Cells were counted on transverse semi-thin sections of utricles from three different experiments for each time point. Their density along a 100-µm length of epithelium is given. (A) Total numbers of supporting cells (Sup. cells), hair cells (total HC) and segregation between identified type I (type I HC), type II (type I HC) and undetermined hair cell type (Und. HC type) were compared with contralateral sections and at 2 h (KA 2 h) and 24 h (KA 24 h) post-lesion formation. Statistical significance between contralateral samples and the 2 h and 24 h time points following one-way ANOVA with post-hoc analysis using the Holm–Sidak method is shown. *P≤0.05, ***P≤0.001. n.s., not significant. (B) The density of identifiable and undetermined hair cell types was analyzed from 1 h to 168 h post-lesion formation in utricles and compared to those in contralateral ears, referenced as time 0 h for statistical analysis. Significance resulting from the ANOVA test followed by the Holm–Sidak method is presented. *P<0.001 vs 0 h, 24 h and 168 h; ^#^*P*<0.001 vs 0 h and 168 h; ^†^*P*<0.001 vs 168 h. Data are mean±s.e.m.

To verify whether the observed kainic-acid-induced vestibular dysfunctions stem from direct damage to vestibular end organs, the tissue was processed for analysis with electron microscopy. The qualitative aspect of the lesion and/or repair was assessed in the utricle on both semi- and ultra-thin sections to investigate the condition of the tissue condition both at cellular and subcellular levels. Figs 2–4 illustrate the early damage observed at the subcellular level 2 h after the STTK injection (Fig 2), the patterns of change in tissue conditions from 2 h to 24 h after the STTK injection (Fig 3) and the quantification of these morphological changes from 1 h to 1 week after the STTK injection (Fig 4). This morphometric analysis was performed on ultra-thin sections. Tissue damage observed in the injured ear was always compared to the intact contralateral ear that did not receive the transtympanic kainate injection. In controlateral tissue, a typical morphology of sensory epithelia containing type I and type II hair cells and supporting cells was observed (Fig. 2A,B; Fig. 3A).

Using electron microscopy, we examined the vestibular sensory epithelia at the cellular and subcellular levels (Fig. 2). Two hours after the STTK injection, typical features of glutamate-elicited excitotoxicity were observed in the injured epithelia (Fig. 2C-F). Some hair cells with their typical afferent terminals were still present: type I hair cells exhibited a pear shape connected by surrounding calyx terminals, whereas type II hair cells displayed a more cylindrical shape and were innervated by classic bouton terminals; however, most of the afferent terminals were very swollen, leaving large vacuoles all along the sensory epithelia (Fig. 2C,D). Membrane disruption of damaged afferent terminals was often observed (Fig. 2E,F). Vacuoles resulting from the swelling of afferent terminals were so extensive that hair cells were distorted and generally no longer identifiable as type I or type II hair cells with regard to their morphology (Fig. 2D-F). Supporting cells were also squeezed, whereas their nuclei remained in the correct position (Fig. 2D,E). No obvious morphological changes of efferent terminals were observed at this time (data not shown). Changes that might have occurred presynaptically were not investigated in the present study.

At the tissue level, the large swellings and induced distortion of the sensory and supporting cells within the epithelium were observed in semi-thin sections (Fig. 3). The quantification of hair cells based on their morphology clearly demonstrated an increase in the number of hair cells for which the specific type I or type II classification could no longer be assigned because of the morphological changes that had occurred 2 h after the STTK injection.

On the basis of histological analysis of vestibular sensory epithelia 2 h after the STTK injection, it can be concluded that the acute peak of altered vestibular behavioral crisis coincided with stereotypical features of excitotoxic damage to afferent terminals within the vestibular epithelia.

### Resorption of swollen afferent terminals in the first 24 h following excitotoxic damage

The utricle, a sensory graviceptor, was observed at the tissue level and quantified at the cellular level in semi-thin sections covering the entire period from 1 h to 1 week after the STTK injection. At the tissue level, the epithelial damage and its progressive resorption were obvious between 2 h and 24 h after the STTK injection (Fig. 3). The time course analysis of the vestibular damage in the utricle at several time points between 2 and 24 h following the STTK injection demonstrated a progressive reduction in the size and number of the swollen afferent terminals (Fig. 3B-F). During that period, despite extensive swelling inside the sensory epithelia, afferent terminals were seen beneath the epithelia in the conjunctive tissue. In semi-thin sections, the kainic acid-induced damage had almost completely resolved 24 h after the STTK injection (Fig. 3F). The general organization of the sensory epithelia appeared normal when compared to that of the contralateral non-injured epithelia (Fig. 3A). Histological analysis of crista ampullaris gross morphology following STTK injection provided results similar to those obtained in utricles (Fig. S1). Altogether, histological observations over the first 24 h following the STTK injection clearly demonstrated a spontaneous resorption of the excitotoxically-induced afferent terminal swelling in the mammalian vestibular sensory epithelia.

In order to specify the time course of the swelling resorption, we performed a morphometric analysis of utricle epithelia over the 24-h period following induction of the excitotoxic insult. The different cell types were identified and quantified using defined morphological criteria to discriminate between supporting cells and hair cells: type I, type II or undetermined. These criteria were based on the localization of nuclei within the sensory epithelium and the hair cell shape. Results are shown in Fig. 4. Over time, no significant loss of supporting or hair cells was detected in the damaged ear when compared to those in the contralateral intact ear (Fig. 4A, data only shown for 2 h and 24 h post STTK). The number of identified type II hair cells in the injured ear did not change between 2 h and 24 h after STTK, but, it was significantly reduced in comparison with that in the contralateral ear. Conversely, the number of identified type I hair cells was significantly reduced in injured utricles 2 h after STTK, although it did not significantly differ from that in intact tissue 24 h after the injury. The number of hair cells of undetermined type increased 2 h after STTK and was still significantly different from that in the contralateral ear 24 h after the injury, consistent with the significant reduction of identifiable type I or II hair cells. Fig. 4B details the time course of the changes in the number of undetermined, type I and type II hair cells between 1 and 24 h after the STTK injection. Quantification at 168 h is also provided for comparison. As early as 1 h after the STTK injection, some hair cells were no longer identifiable as type I or type II hair cells. At this time, the number of type II hair cells had already significantly decreased to its minimum and the number of type I hair cells started to decline whereas the number of hair cells of undetermined type conversely increased. From 2 to 12 h, a maximal and steady quantity of hair cells of undetermined type was reached. At that time, the number of cells identified as type I hair cells was minimal. At 12 h after the STTK injection, the number of identified type I hair cells drastically and statistically increased. Conversely, the number of hair cells of undetermined type started to decline. At 24 h post STTK, the quantity of hair cells exclusively identified as type I was similar to that in the contralateral non-injured ear. Both the numbers of type II and undetermined type hair cells remained statistically different. One week (168 h) after the STTK injection, the numbers of identifiable type I and type II cells, and of cells of undetermined type were similar to those in the contralateral ear. Taken together, these results demonstrate that spontaneous resorption of swollen afferent terminals occurred in the injured vestibular epithelia after kainic acid-induced excitotoxic damage following a specific time course. The resorption process resulted in a general restoration of sensory and supporting hair cell organization. However, to determine the degree of damage and repair of terminal afferents at the sub-cellular level, a more precise analysis using electron microscopy was required.

### Repair of afferent terminals throughout the first week following excitotoxic insult

**Fig. 5.**
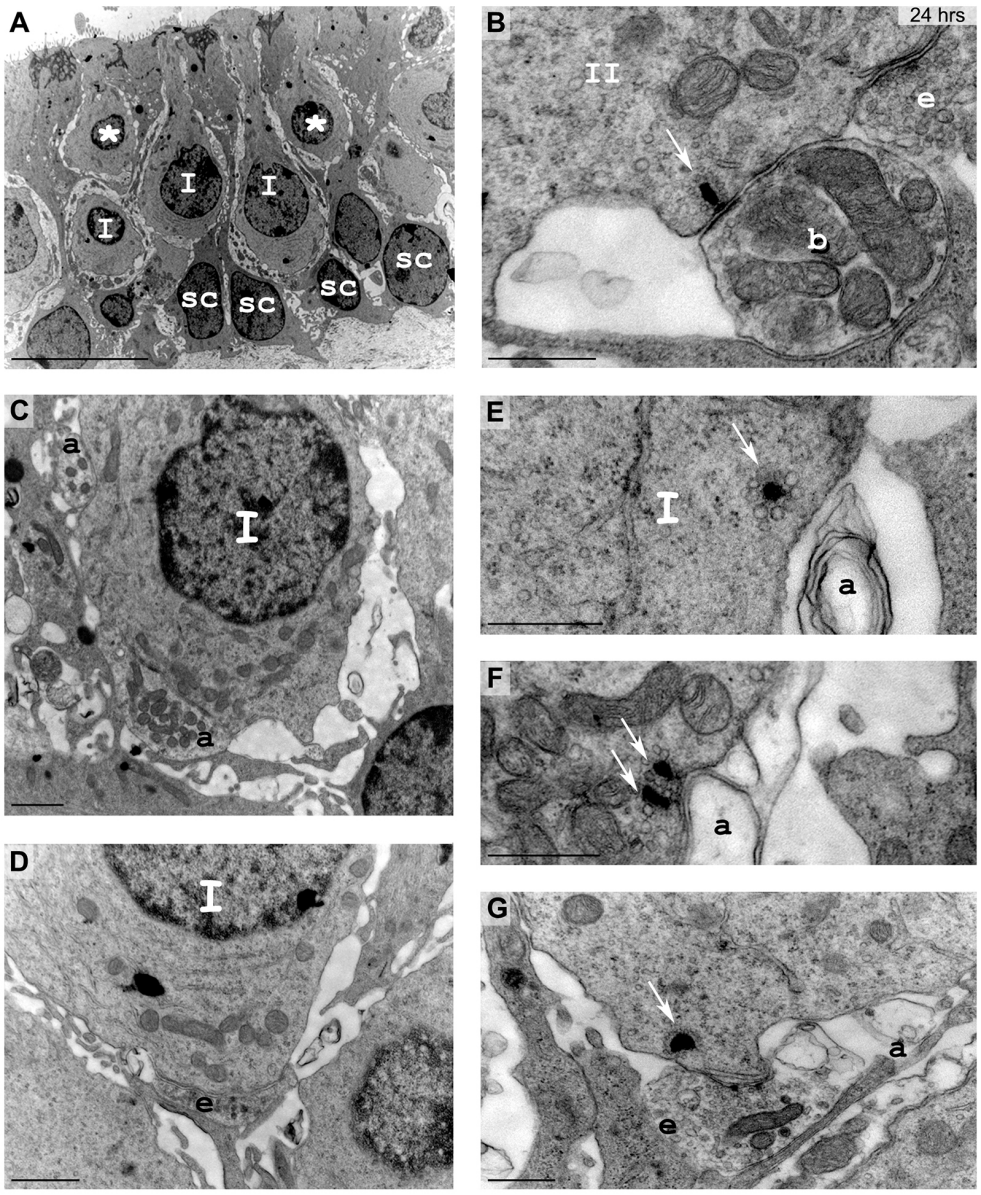
**Detailed morphology of vestibular epithelia 24 h after the excitotoxic injury.** Utricles were observed with electron microscopy. (A) At low magnification, some type I hair cells (I) were identified, whereas other hair cells with an undetermined type remained (*). Sc, supporting cells. (B-G) Nerve terminals were detailed at higher magnification. (B) Typical ribbons (white arrow) facing postsynaptic densities on bouton afferent fibers were observed. II, type II hair cells. (C,D) Bouton-like terminals (a) or efferent terminals (e) unusually connected some type I hair cells. (E,F) Single or multiple ribbons (arrows) faced remaining pieces of calyceal membrane. (G) Typical features of competition between efferent terminals and afferent fibers to contact hair-cell-facing ribbons (arrow) were found. Scale bars: 10 µm (A); 1 µm (C,D); 500 nm (B,E-G).

As previously stated, the overall organization of the sensory epithelium appeared to be properly restored within the first 24 h following the kainate injury when observed using light microscopy analysis of semi-thin section preparations (see Fig. 3). However, this level of observation did not provide enough detail to be able to assess the precise stage of the repair at the subcellular level, particularly when substantial vestibular dysfunction persisted (Fig. 1). To address this point, we used electron microscopy to examine the contacts between hair cells and their associated afferent terminals in rat utricles 24 h after STTK injection (Fig. 5). Compared to non-damaged tissue, most type I hair cells were easily identified according to their pear-shaped morphology, constricted neck and the basal localization of their nuclei (Fig. 5A). Type II hair cells were often contacted by bouton afferent terminals that normally faced ribbons and by efferent terminals filled with synaptic vesicles facing synaptic cisterns (Fig. 5B). No further swelling was observed. However, close examination of type I hair cells and their associated calyx terminals revealed unusual features of altered contacts (Fig. 5C-G): some terminals were simply absent, whereas other terminals displayed features of immature terminals (Desmadryl and Sans, 1990). Some type I hair cells were surrounded by fragmented calyx terminals (Fig. 5C). Single (Fig. 5E) or multiple (Fig. 5F) ribbons were found to face remnant membranes of calyx terminals that no longer possessed any mitochondria. The most striking feature was the presence of efferent terminals, full of vesicles with small mitochondria that directly contacted the type I hair cells (Fig. 5D,G). In some cases, efferent terminals appeared to compete with afferent terminals in front of presynaptic ribbons (Fig. 5G). It is noteworthy that a similar innervation pattern is usually observed during development (Desmadryl et al., 1992). Quantification of type I hair cells (*n*=117) and their calyx terminal afferents revealed that only 26.33±0.85% (mean±s.e.m.) of type I hair cells were contacted by fully formed calyces 24 h after the excitotoxic injury. The others were contacted by either partial calyx terminals (32.60±7.44%) or not contacted at all (41.07±6.63%).

**Fig. 6.**
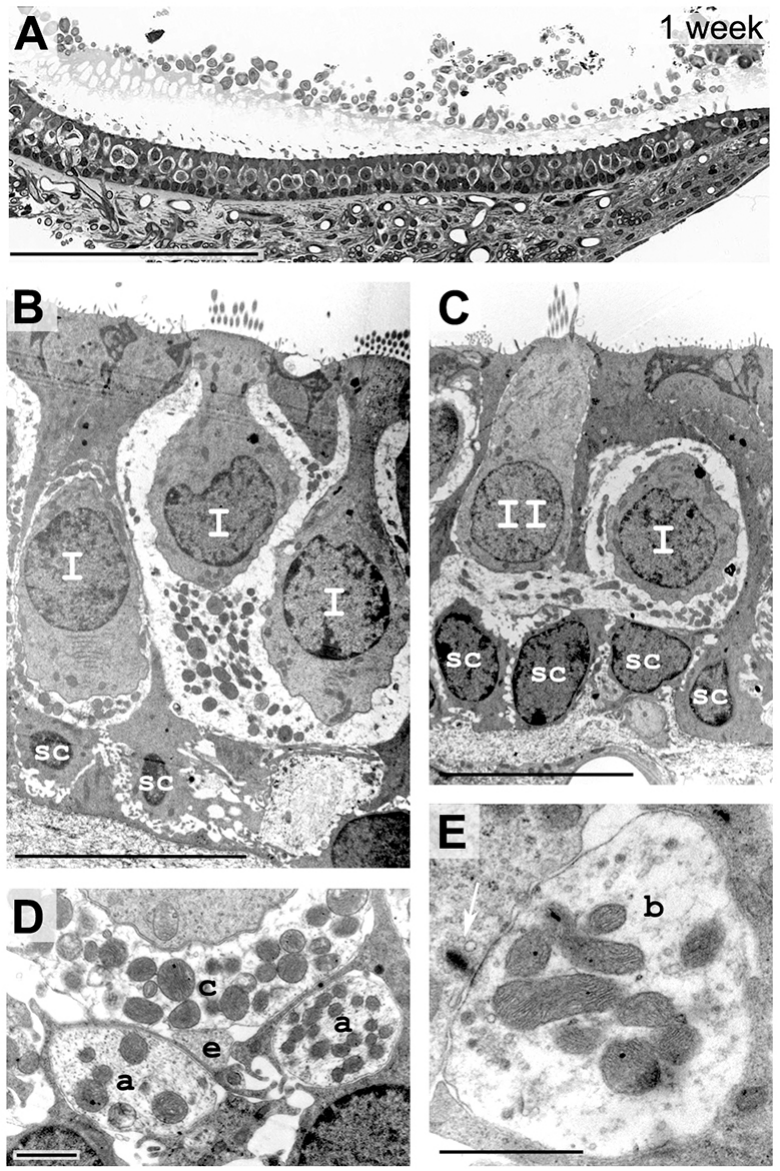
**Histology of sensory epithelium 1 week after excitotoxic injury.** (A) Transverse semi-thin sections of the injured utricle. At 168 h, the lesion shows a normal gross morphology and organization of hair cells and supporting cells, beneath the conjunctive tissue where innervating fibers were observed. (B,C) Electron microscopy shows that morphology of type I (I) and type II (II) hair cells was normal; terminal afferents are present. (D,E) At higher magnification, regular afferent terminals were observed: calyx connect type I hair cells (c), efferent terminals contact calyx afferents (e), afferent fibers are passing (a) and bouton afferent terminals (b) contact facing ribbons (white arrow) of type II hair cells. Scale bars: 100 µm (A); 10 µm (B,C); 1 µm (D,E).

One week after the kainic acid induction of excitotoxic damage, vestibular tissue was analyzed to assess the degree of completion of the repair process. On semi-thin sections (Fig. 6A), the gross morphology of sensory epithelia did not significantly differ from contralateral non-injured ear tissue (for quantification of hair cells, see Fig. 4B). At the ultra-structural level, the detailed morphology also appeared normal. Type I and type II hair cells were clearly identifiable (Fig. 6B-C) with proper calyx (Fig. 6D) or bouton (Fig. 6E) afferent terminals connected.

### Replenishment of afferent terminals with synaptic vesicles

**Fig. 7.**
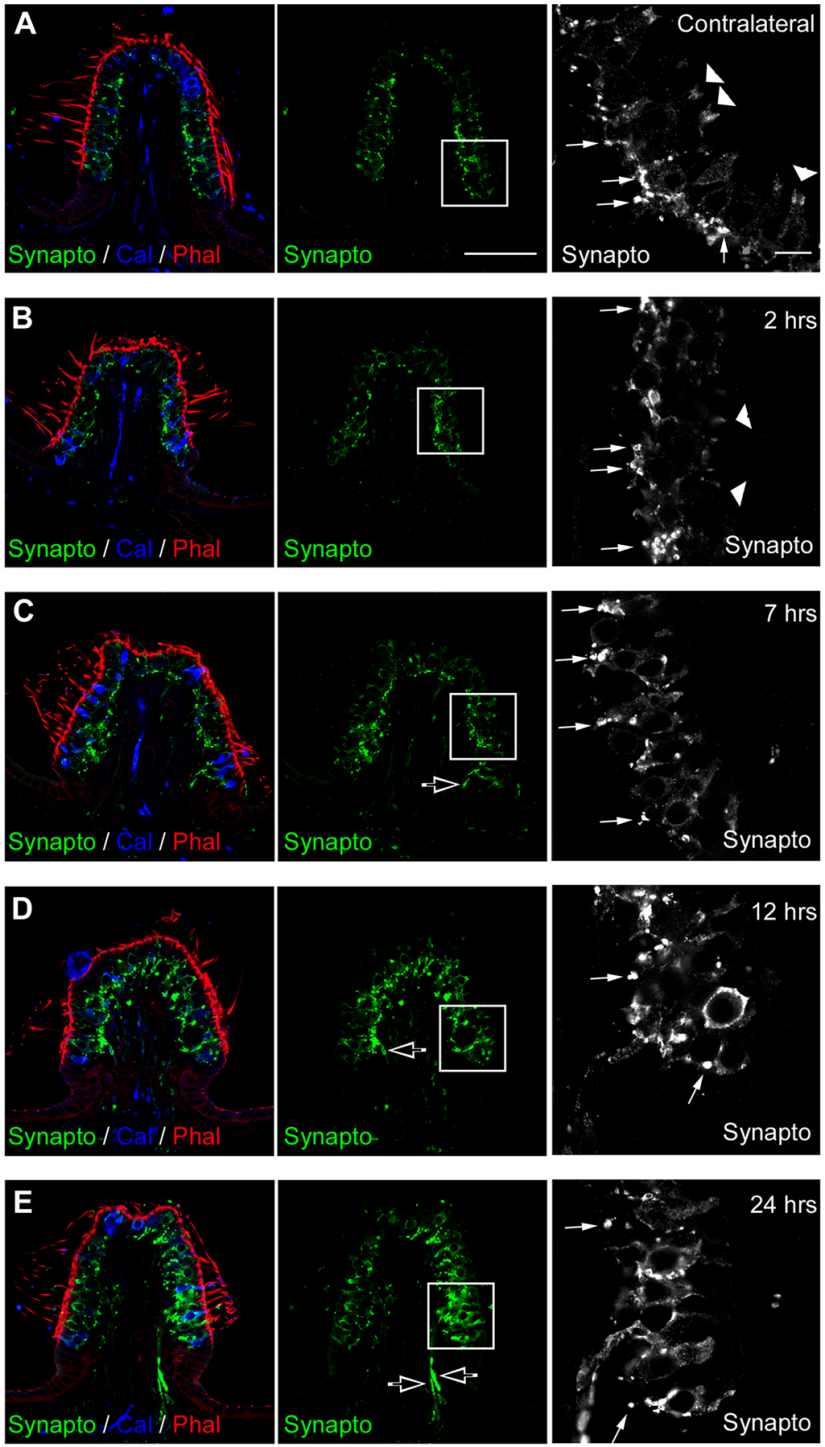
**Progression of synaptophysin expression over time after excitotoxic injury.** Immunolabeling in a transverse section of crista observed by confocal microscopy. (A) In the control ear, counter-labeling of transverse sections with phalloidin (Phal, red) and antibodies against calretinin (Cal, blue) highlight hair bundles of sensory cells, and most type II hair cells and pure calyx of the central zone. Immunolabeling with antibodies against synaptophysin (Synapto, green and white) locates small synaptic vesicles in normal vestibular sensory epithelium. High magnification of the inset (black and white panel) shows precisely that synaptophysin is expressed in efferent terminals (arrows) and at the top of calyces (arrowheads) in this adult tissue. (B-E) Between 2 h and 24 h after the STKK injection, gross morphology of the sensory epithelium was distorted, as evidenced by the actin labeling, and synaptophysin expression changed. (B,C) At 2 h and 7 h, synaptophysin became mostly disorganized, with loss at the top of calyces. (D,E) Between 12 h and 24 h, expression increased in innervating fibers within the conjunctive tissue (open arrows, C-E) and hair cells. Scale bars: 10 µm.

**Fig. 8.**
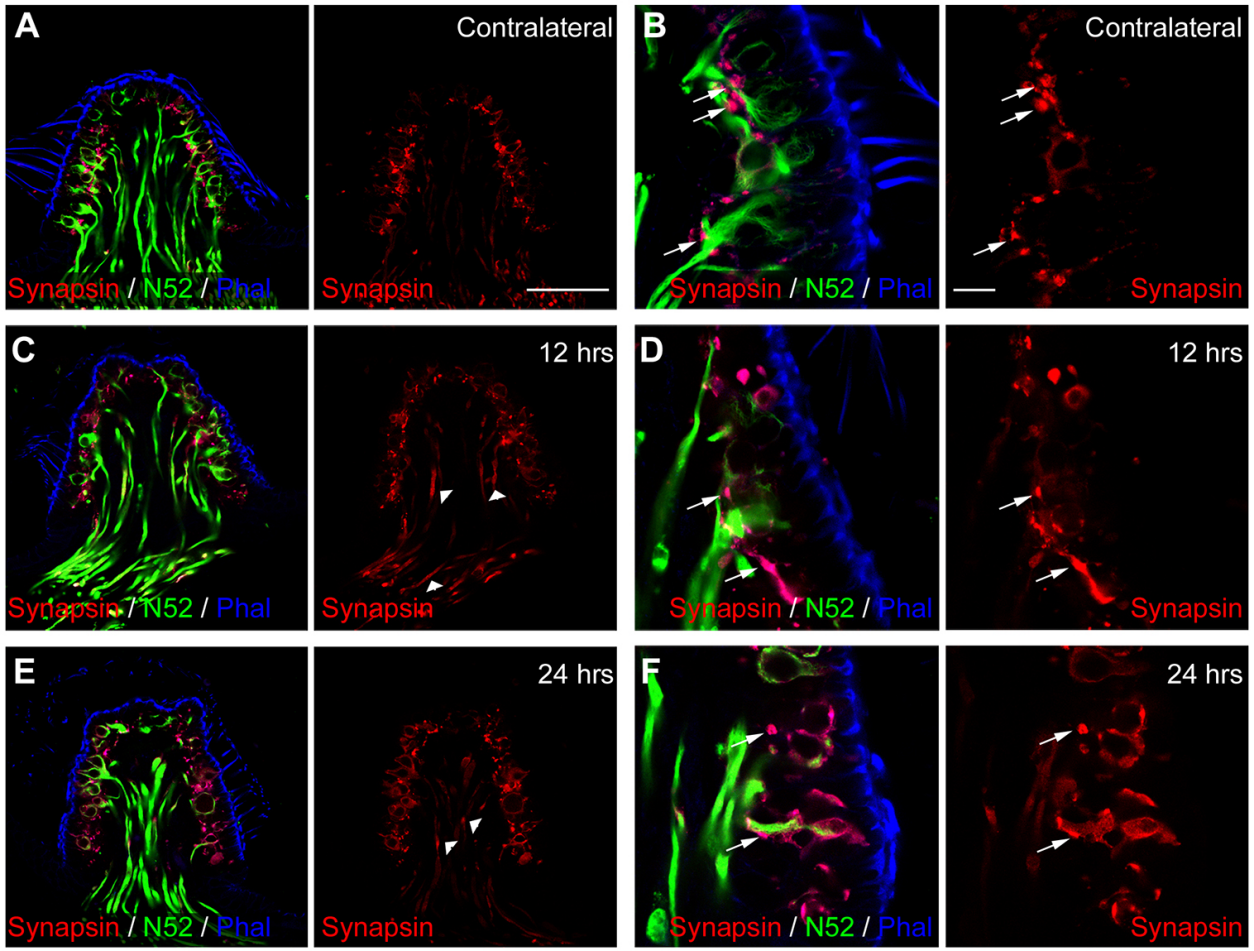
**Progression of synapsin expression over time after excitotoxic injury.** Immunolabeling in transverse sections of crista observed by performing confocal microscopy. (A,B) In the contralateral ear, counterlabeling of transverse sections with phalloidin (Phal, blue) and antibodies against neurofilament 200 kDa (N52, green) highlight hair bundles of sensory cells and afferent fibers. Immunolabeling with antibodies against synapsin (Synapsin, red) locates small synaptic vesicles in normal vestibular sensory epithelium. (B) High magnification shows precisely that synapsin is expressed in efferent terminals (arrows) contacting afferent fibers. (C,D) At 12 h after the STTK injection, synapsin expression became disorganized and increased. (E,F) At 24 h after the STTK injection, synapsin was also expressed in afferent terminals (arrowheads). Scale bars: 10 µm (A, applies to C,E, and B applies to D,F).

To further investigate the process of hair cell re-innervation following excitotoxic damage to vestibular afferents, we monitored the expression of synaptophysin (Fig. 7) and synapsin (Fig. 8), two proteins associated with synaptic vesicles (Brachya et al., 2006), in cristae at 2 h, 7 h, 12 h and 24 h after the STTK injection. In contralateral non-injured sensory epithelia (Fig. 7A), immunolabeling against synaptophysin revealed dot-like structures that were restricted to the base of the sensory cells (Fig. 7A, arrows) and the apex of the epithelia (Fig. 7A, arrowheads). This labeling matched previously described expression of synaptophysin in both the efferent and afferent bouton terminals, as well as at the upper portion of the calyx afferent terminals reported in the adult rodent vestibule (Dechesne et al., 1997; Sage et al., 2000; Scarfone et al., 1988, 1991). At 2 h after the excitotoxic insult, synaptophysin expression did not differ from the contralateral ear, except for a slight disorganization at the base of hair cells and at the apex of calyces (Fig. 7B). The lack of synaptophysin expression in the conjunctive tissue underlining the cristae up to 7 h after the STTK injection is noteworthy. At 7 h after the excitotoxic injury, synaptophysin immunoreactivity started to appear in some afferent fibers entering the sensory epithelia as well as in hair cells. Subsequently, immunolabeling increased during the proceeding hours in both the afferent terminals and in hair cells (Fig. 7D-E). Of note was the entrance of fibers that intensively expressed synaptophysin 24 h after the STTK injection (Fig. 7E, open arrows). This alteration of the synaptophysin relocation and intensification confirmed the re-organization of nerve terminals, mimicking the processes observed during the developmental synaptogenesis period (Dechesne et al., 1997; Gaboyard et al., 2003; Sage et al., 2000; Scarfone et al., 1991). Synapsin expression was more specific to efferent terminals only in the adult vestibule, as we observed in the contralateral ear (Fig. 8A,B). Synapsin expression was mostly restricted to dot-like structures at the base of sensory cells that lined but clearly did not overlap or coincide with the neurofilament staining (Fig. 8B, arrows), corresponding to localization of mature efferent terminals (Favre et al., 1986; Holstein et al., 2005). At 12 h after the excitotoxic damage, synapsin immunoreactivity increased in the cristae, with a reorganization of efferent terminals, and expression of synapsin increased in fibers entering the epithelia and in hair cells (Fig. 8C-F, arrowheads). A similar pattern of expression of these proteins, which are associated with synaptic vesicles, has been described during the process of perinatal synaptogenesis in the vestibule (Scarfone et al., 1991).

## DISCUSSION

The present study addresses the lack of information regarding the different stages and time course of damage and repair processes at inner ear nerve terminals following excitotoxic damage. Adapted to vestibular afferents, this work is crucial to understand how physical damage at the contact between sensory hair cells and nerve terminals arising from the primary vestibular neurons governs the heterogeneity of symptoms of vestibular disorders.

### Inducing and assessing excitotoxic damages in the mammalian vestibule

The term ototoxicity applies to specific damages induced by the application of different drugs to auditory or vestibular sensory cells. Vestibular hair cell loss is a typical pathophysiology induced by aminoglycosides, such as gentamicin, or nitriles such as 3,3′-iminodipropionitrle (IDPN). Recent studies have demonstrated that in addition to hair cell impairment, the calyx innervation of type I hair cells is also altered by gentamicin or IDPN administration, suggesting that excitotoxicity and damage to the afferents might also be involved in the pathophysiology of drug-induced ototoxicity (Hirvonen et al., 2005; Sedo-Cabezon et al., 2015). Finally, the sole correlation between structural excitotoxic damages within the vestibule and altered vestibular behavior has rarely been studied. Excitotoxic injury of vestibular afferents has been previously undertaken using different delivery methods. Surgical approaches, syringe pumps and gelfoam containing glutamate agonists have been used to deliver excitotoxic drugs in the immediate proximity of the vestibule (Brugeaud et al., 2007; Liu, 1999; Shimogori and Yamashita, 2004). In comparison to the currently used transtympanic injection, these delivery methods were supposed to reduce the dosage and exposure time necessary for the excitotoxic agents to have an effect. However, none of the extensive histological damage that we observed in the present study (i.e. large vacuoles in place of afferent terminals with distortion of hair cells) has been described in the previous studies. Reasons for the variable severity of excitotoxic damages might include, firstly, a difference in the effective dose of drug that reaches the inner ear. Liu (1999) used a direct application but of only small drops with a concentration of 10 to 50 nM kainic acid. Brugeaud et al. (2007) delivered a higher dose of 5 mM kainic acid but with extended diffusion over time from a gelfoam placed against the round window, probably resulting in longer exposure but a lower peak dose. The time points of histological exploration could also have played a role in the observed degree of damage severity. Through their early analysis of excitotoxic damage, performed 15 min following the kainic acid application, Liu (1999) could have missed the maximum damage that we observed in the present study 1.5 h after the kainic acid injection. Shimogori and Yamashita (2004) have investigated vestibular epithelia as late as 1 week after the injury (when we generally observed complete macroscopic recovery), and the histological method used was only light microscopy without the resolution necessary to precisely observe synaptic injury. One recent study using transtympanic injections of kainic acid (Dyhrfjeld-Johnsen et al., 2013) resulted in vestibular dysfunction recorded with both behavioral testing and electronystamography. Damaged afferents were clearly observed 24 h and 48 h after the insult, but initial tissue lesions following the excitotoxic induction during the early stages was not described.

Regarding the behavioral assessments, the vestibular deficit induced by Brugeaud et al. (2007) was quite similar to those we observed 48 h after induction. However, they did not observe a pronounced behavioral peak crisis as we did in the present study (personal communication), suggesting that the delivery method might not have induced immediate strong lesions similar to those obtained through transtympanic injection. Conversely, Shimogori and Yamashita (2004) observed AMPA-induced vestibular disorders immediately following the excitotoxic induction. They report strong spontaneous nystagmus during the first 12 h post injury that could correspond to our initial peak in damage at 12 h. In summary, the results of those different studies compared to present results strongly suggest that (1) selective damage to the vestibular afferents directly supports the altered behavior characteristics of vestibular deficit, (2) the degree of structural damage (severity and duration) is directly reflected by the severity of vestibular deficit, and (3) following vestibular damage and deficit, structural repair and functional recovery occur.

### Excitotoxic features at vestibular afferents during acute peak crisis

Transtympanic injection of kainic acid induced vestibular dysfunctions corresponding to a moderate behavioral disorder score (Brugeaud et al., 2007; Desmadryl et al., 2012; Dyhrfjeld-Johnsen et al., 2013; Vignaux et al., 2012). We report three sequential stages, starting with a pronounced acute crisis during the two first hours after the insult, followed by a relatively high and stable vestibular dysfunction period lasting several hours and a subsequent slow functional recovery period that is completed within several days. The acute crisis coincides with histological damage stereotypical of excitotoxicity within the vestibular end organs. Large swelling of afferent terminals that could already be observed 1 h after the insult induction persisted for a 7-h period, correlating with the duration of the acute crisis. Such extensive swelling of afferent terminals with hair cell distortion has been previously described in the cochlea, following noise-induced trauma or models of excitotoxicity with glutamate receptor agonists (Pujol and Puel, 1999; Ruel et al., 2007), and in both cochlea and vestibules following ischemia (Puel et al., 1994; Sasaki et al., 2012). Furthermore, in the central nervous system, ischemia and local or systemic injection of glutamate receptor agonists induce swelling of afferent terminals (Nikonenko et al., 2009; Schwob et al., 1980; Sperk et al., 1983). This trauma has been referred to as an early stage of excitotoxicity. *In vivo* and *in vitro* experiments have been used to demonstrate that this mechanism mainly depends on non-NMDA receptors and results from massive influx of Na^+^, Cl^−^ and water into the afferent terminals (Choi, 1987; Hoyt et al., 1998). Depending on the cause and duration of the insult, subsequent death of the injured neurons might occur through apoptotic and necrotic pathways (Gwag et al., 1997; Koh et al., 1990; Munir et al., 1995). The balance between neuronal repair and neuronal death after excitotoxicity has been well described in the cochlea and basilar papilla of birds using sustained and/or higher doses of glutamate receptor agonists (Sun et al., 2001).

In the present study, we show that the slow and partial functional recovery of balance function coincides with a subsequent stage of retraction of the afferent terminals occurring within the first 24 h after the excitotoxic insult. The retraction terminates either with partial loss of the calyx apposition or with a complete ‘stripping’ of the hair cell basolateral membrane. Features of the retraction phase remained visible within the epithelia up to 24 h under the present paradigm. This retraction mechanism seems to mainly affect the distal unmyelinated part of the nerve terminals of Scarpa's ganglion neurons. Working on the severity of this excitotoxic model, modifying the dose and number of transtympanic administrations of kainic acid for example, will help to answer some fundamental questions concerning the degree of damage and the capacity for repair (Tos et al., 2013).

### Repair of vestibular afferent terminals mimics developmental processes

In the present study, swelling process and resorption of afferent terminals correspond to an acute episode of vestibular disorder with pronounced behavioral dysfunction. Subsequently, we observed the retraction of nerve terminals followed by the spontaneous repair of afferent terminals that starts 12 h after the excitotoxic insult and coincides with a slow functional recovery of the balance function. Interestingly, the step of spontaneous repair displays features previously observed in mammalian vestibules during the perinatal synaptogenesis period (Dememes et al., 2001; Desmadryl et al., 1992; Desmadryl and Sans, 1990). Competition between afferent and efferent terminals for innervation of type I hair cells, with direct apposition of efferent terminals on type I hair cells, were often observed. Synaptophysin and synapsin are both required for neurotransmission, and are involved in synaptogenesis and neuronal plasticity (Brachya et al., 2006; Cesca et al., 2010). During maturation of vestibular end organs, synaptophysin and synapsin switch from a pre-synaptic and diffuse expression in hair cells with post-synaptic expression along entering fibers to a precise location at the apex of calyces, and in afferent and efferent terminals in adults (Dechesne et al., 1997; Favre et al., 1986; Gaboyard et al., 2003; Holstein et al., 2005; Scarfone et al., 1988, 1991). In the present study, expression of synaptophysin and synapsin increased 12 h after the excitotoxic insult, and their expression appeared to mimic that which has been previously described during development, with increased labeling of afferent fibers and terminals. Our observation confirmed that afferent repair that occurred after excitotoxic damage appeared to engage mechanisms that are observed during the development of this sensory organ (Brugeaud et al., 2007). As supposed for other systems during repair plasticity (Hou and Dahlstrom, 2000; Marti et al., 1999), the increased expression of synaptophysin and synapsin could reflect a process of replenishment of small synaptic vesicles to re-establish lost terminal contacts. Another role for the increased expression of these proteins in calyx terminals is likely to be their participation in the process of membrane resealing. This mechanism, which has been previously described for synaptobrevin or SNAP25 (Steinhardt et al., 1994), would indeed be a good counter measure against the destruction of the membrane during the swelling process.

**Fig. 9.**
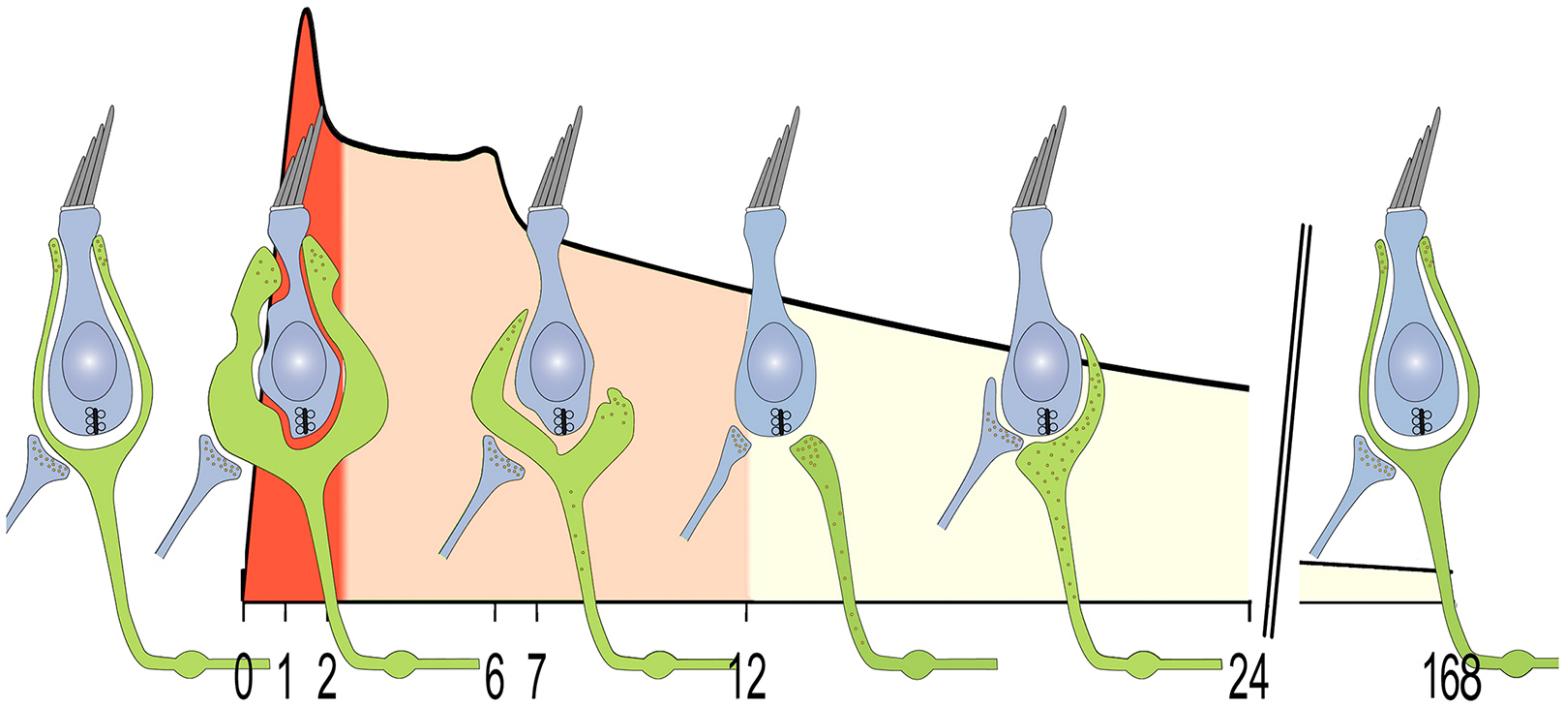
**Illustration of the correlation between vestibular dysfunction and changes at the vestibular afferent terminals.** Diagram shows the time course of behavioral vestibular dysfunctions induced by transtympanic injection of kainic acid (curve) and the change occurring at calyx terminals after excitotoxic injury (schematic cartoon of hair cells and afferents). The acute crisis peaking at 1.5 h followed by the progressive recovery period of the vestibular function parallels the lesion (red), terminal resorption (orange) and repair (yellow) of the afferent terminals. Replenishment of proteins associated with small synaptic vesicles is shown.

Taken together, these results confirm and strengthen previous observations that spontaneous repair of vestibular afferent terminals occurs in mammalian vestibular sensory epithelia following excitotoxic damage (Brugeaud et al., 2007; Dyhrfjeld-Johnsen et al., 2013). The repair process follows a precise time course that has already started 24 h after the excitotoxic injury and is completed within 1 week. Its sequence follows successive and identified steps: swelling, resorption and retraction of nerve terminals followed by competitive re-innervation and *de novo* formation of afferent terminals (Fig. 9). The re-innervation process mimics the process encountered during developmental synaptogenesis.

### Capacities for and limits of functional recovery

The structural repair was accompanied by functional recovery of the vestibular behavior. As previously stated, various studies have explored over the last decades the mechanisms of inner ear damage and repair using different paradigms to mimic pathological conditions in the auditory organ. Animal models of excitotoxic lesions through local application of glutamate receptors agonists, noise trauma and ischemia has enabled us to decipher the correlation between damage to the auditory synapses and hearing loss (for review see Pujol and Puel, 1999; Ruel et al., 2007). Spontaneous repair of afferents has been reported in the cochlea, and this synaptic plasticity is accompanied by recovery of the auditory function. Recently, the repair capacities have been challenged through strong auditory trauma that induces synaptopathy or neuronal death, resulting in partial but irreversible loss of auditory function (Kujawa and Liberman, 2009; Liberman and Liberman, 2015; Lin et al., 2011).

In the vestibule, no deleterious effect of over stimulation has ever been reported. Conversely, ischemia has been demonstrated to act as a potential source of damage (Jiang and Umemura, 1993; Kitamura and Berreby, 1983; Nario et al., 1997; Oas and Baloh, 1992; Ritter and Veit, 1977; Rubinstein et al., 1988; Tange, 1998). Better protection and repair might also occur in the vestibular end organs because major differences in the branching patterns of connections exist between the sensory organs. Primary vestibular neurons display multiple connections to both type I and type II hair cells (for recent review see Eatock and Songer, 2011), whereas cochlear primary neurons display a single connection to only one inner hair cell (for recent review, see Kujawa and Liberman, 2009). The redundancy of connections in the vestibule might promote higher protection than in the single-branched cochlear neurons. The severity of damage and the degree of fiber retraction beneath the sensory epithelium might condition the capacity of nerve fibers for regrowth and reconnection (Tos et al., 2013). In the present study, and previous ones, using the kainic acid paradigm (Brugeaud et al., 2007; Desmadryl et al., 2012; Dyhrfjeld-Johnsen et al., 2013), we demonstrated that the severity and duration of the acute vertigo crisis correlate with the extent of the insult to afferent terminals. It can be assumed that too much damage and retraction of the afferent terminals compromises the capacity for repair. A massive release of glutamate during sustained ischemia or prolonged excitotoxic conditions could cause massive synapse uncoupling and pronounced retraction that would further delay, and ultimately prevent, any possibility of regrowth and reconnection. Such a situation might reflect the clinical tableau sometimes encountered in humans in cases of complete vestibular areflexia as a result of conditions diagnosed as labyrinthitis or vestibular neuritis, or in cases of cophosis encountered in sudden sensory hearing loss conditions (Moalla et al., 2007). Further quantitative analysis will be needed to precisely establish the capacity for plasticity of vestibular synapses exposed to different traumatic conditions. The present model of kainic-acid-induced excitotoxicity might be a good starting tool for further investigation of such questions by performing some experiments involving dose responses or modified durations of exposure to the glutamate agonists. Also, quantitative assessment of the presynaptic proteins should yield information on the state of the presynapse during the excitotoxic process.

In birds, destruction of hair cells by ototoxic agents induces a complete regeneration of the sensory epithelia with a full set of sensory cells and complete re-innervation (Zakir and Dickman, 2006). However, the branching pattern of the regenerated synaptic connections is modified and correlates with alterations in gaze responses (Haque et al., 2009). In response to peripheral impairment of the vestibule, such as rebranching, re-innnervation or destruction, a central compensatory mechanism restores balance function (Yu et al., 2014). One can ask how a central nervous system mechanism is executed to support vestibular compensation during this short period of lesion and repair. This central process has to deal with and balance both the early stages of the insult, inducing a strong alteration of the peripheral information, and the subsequent and progressive spontaneous restoration of the peripheral vestibular afferents, re-introducing a modified sensory signal. A better understanding of what happens in terms of reactional recomposition within the brain stem vestibular nuclei, depending on the type of peripheral damage, will probably provide answers to this question (Lacour et al., 2009).

In conclusion, this study provides, for the first time, precise information on the sequence of the early events that occur following an excitotoxic insult within the vestibular end organs. We have detailed the structural changes that occur at vestibular afferent terminals and their correlation with symptoms of vestibular dysfunction. Going further will require identifying the molecular actors involved and determining the correlation between the severity of the insult and spontaneous repair capacities. This information is a prerequisite for further development of targeted pharmacological approaches to efficiently protect vestibular afferent terminals and/or promote their regeneration.

## MATERIAL AND METHODS

### Animals

Experiments used adult female Wistar rats (2 months of age, 200-224 g; CERJ, Le Genest, France) in accordance with the French Ministry of Agriculture regulations and European Community Council Directive no. 86/609/EEC, OJL 358.

### Unilateral vestibular excitotoxic insult

Using a surgical microscope, single transtympanic injection of the glutamate receptor agonist kainic acid (referred as STTK, 12.5 mM, 100 µl; Ascent Scientific, Bristol, UK; *n*=29) was performed under isoflurane anesthesia according to the procedure previously detailed (Dyhrfjeld-Johnsen et al., 2013). In summary, rats were anesthetized with isoflurane and kept in a lateral position. Applying traction to the auricle, the posterior portion of the tympanic membrane was penetrated with a sterile insulin syringe (0.33×12.7 mm or 0.5×16 mm). Kainic acid, dissolved in 100 µl of saline, was slowly injected into the middle ear cavity. After injection, the animal was kept in a lateral position for 10-15 min under isoflurane anesthesia, and observed until it had recovered from the anesthesia.

Control animals (*n*=8) were also subjected to transtympanic injection into the right middle ear of only saline solution.

### Behavioral experiments

A vestibular dysfunction score was determined as previously described (Boadas-Vaello et al., 2005; Brugeaud et al., 2007). Altered vestibular behaviors were scored on a scale from 0 to 4, ranging from normal behavior (rating 0) to maximal identified vestibular impairment (rating 4). Table S1 describes the observed behavior and associated criteria used to score the vestibular dysfunction (normal=0; mild deficit=2; maximal and severe dysfunction=4). The six different tests were sequentially scored and summed to rate the vestibular dysfunction: 1, head bobbing (abnormal intermittent backward extension of the neck); 2, circling (stereotyped movement ranging from none to compulsive circles around the hips of the animal); 3, retro-pulsion (typical backward walk reflecting vestibular disturbance); 4, tail-hanging reflex (normally induces a forelimb extension to reach the ground, unilateral dysfunction results in axial rotation of the body); 5, contact-inhibition reflex (normally leads animal to release hold grip on metal grid in a supine position when their back touch the ground; in case of vestibular dysfunction with lack of full body orientation reference, animal retains grip on the grid in a supine position); 6, air-righting reflex (necessary for rats to land on all four feet when falling from a supine position; vestibular dysfunction impairs normal body repositioning with a maximal dysfunction leading the animal to land on its back when dropped from a height of 40 cm onto a foam cushion). Rats were scored for vestibular dysfunctions just before the STTK injection and evaluated 1, 1.5, 2, 3, 5, 7, 24, 48, 72, 96 and 168 h after the injection (Fig. 1). Scores were expressed as mean±s.e.m.

### Tissue preparation for histology

Animals were deeply anesthetized with pentobarbital (100 mg/kg), then perfused transcardially with heparin PBS (0.01 M) followed by a fixative solution (2% paraformaldehyde, 2.5% glutaraldehyde, 1% picric acid, with 5% sucrose for light and electron microscopy; 4% paraformaldehyde, 1% picric acid, with 5% sucrose for immunolabeling). Temporal bones were postfixed in the same solution before vestibular epithelia were dissected in PBS. Tissue from the right injected ear and control naive left ear were carefully identified and segregated.

### Light and electron microscopy

Whole fixed vestibular organs from animals perfused at 2, 5, 7, 12, 24 or 168 h after the STTK injection were postfixed in 0.5% OsO_4_, dehydrated and embedded in Araldite. For light microscopy analysis, semi-thin (1 µm) sections were systematically cut with an ultra-microtome (Ultracut, Reichert-Jung). To quantify hair cells within utricles and ensure comparative unbiased counts between the different time points, we determined a pre-established pattern for sectioning and sampling. Sensory epithelia were embedded in resin with a longitudinal alignment to obtain transverse sections from the first-third part of the utricle following an antero-posterior orientation (Desai et al., 2005b). Transverse semi-thin sections were then cut within each sample. When hair cells were first observed in sections, 100 µm of epithelium was cut before collection for quantification. Then, five sets of three serial sections were collected, each set was separated by 25 µm. Semi-thin sections were Toluidine-Blue stained, mounted on slides and scanned using the Nanozoomer slide scanner (objective 40×; Hamamatsu, Japan). For electron microscopy analysis, ultra-thin sections (80-90 nm) were cut at 2, 24 and 168 h post STTK. Five sections were cut and collected after each set of semi-thin sections. They were stained with uranyl acetate, then viewed and digitalized in a Hitashi H7100 microscope.

### Morphometry

For quantification of hair cells and supporting cells within utricles, Metamorph software (Molecular Devices, Sunnyvale, CA) was used and cells were manually counted on semi-thin sections. Collection of sections starting 100 µm from the anterior edge of the sensory epithelium ensured a sampling of a transverse section composed of both a striolar and extrastriolar region (Desai et al., 2005b). The 25-µm spacing between each set prevented counting of the same hair cell twice in two distinct sets of serial sections (mean hair cell width=10 µm, calculated from Desai et al., 2005b). Three serial sections were collected for each set of transverse sections of epithelium to ensure acquisition of one full section per set suitable for quantification. For each utricle, three sections collected from three different sets were quantified. Thus, a snap shot of around 400 hair cells, sampled within the first third of each utricle and coming from both striolar and extrastriolar regions, were counted for each time point from three different animals. Counts from the contralateral ears were pooled as control. Cells were classified based on morphological criteria; cell shape and position of the nucleus were the main ones. The following classifications were used: (1) type I hair cell, when a pear-shaped hair cell was observed with basal nucleus localization; (2) type II hair cell, when an elongated hair cell with a nucleus positioned high in the cell was observed; (3) undetermined hair cell type, when the swollen terminal around the cell prevented determination of the cell type based on its shape, or when an elongated cell had a nucleus in a more basal position; and (4) supporting cells, forming the basal part of the epithelium. Note that calyx innervation is a classic criterion used to identify type I hair cells; however, our observations at this magnification prevented clear-cut identification of afferent terminals. Thus, we preferred avoiding the presence of calyx innervation as an identification criterion and only used it as a confirmatory criterion for hair cell type I classification when it was definitely observed. The number of counted cells was standardized to the length of the analyzed transverse section.

For quantification with electron microscopy, the aim was to quantify type I hair cells and their afferent terminals. Thus, only hair cells identified as type I (*n*=117) by their pear shape and low-level nucleus were analyzed on ultra-thin sections from three different animals fixed 24 h after STTK. Quantification was performed on three ultra-thin sections collected from three different sets to obtain a snapshot of type I hair cells and calyx innervation from striolar and extrastriolar regions without overlapped counts. They were categorized and counted depending on the state of their calyx afferent innervation: (1) full if reaching their neck, (2) partial if present but not fully formed and (3) absent if lacking.

### Statistical analysis

SigmaPlot Software (Systat Software, Chicago, IL) was used to perform appropriate statistical tests. For behavioral scoring, Friedman repeated measures analysis of variance (RM-ANOVA) was followed by Dunn's method to analyze the vestibular dysfunction over time after kainic acid injury. For comparison to saline control animals, the Mann–Whitney Rank Sum Test was used. For histological analysis, one-way analysis of variance (one-way ANOVA) was followed by the Holm–Sidak method to determine significant differences in cell counts for the different time points. Comparisons over time, with contralateral ears referenced as time 0 h, were performed from 0 h to 168 h for each type of hair cell.

### Immunochemistry

Vestibular end organs from animals perfused at 2, 7, 12 or 24 h after the STTK injection were embedded in 4% agarose, and 40-μm-thick sections were cut with a vibratome instrument (HM650V, Microm). The free-floating sections were first permeabilized with 4% Triton X-100; non-specific binding was prevented by a pre-incubation step in a blocking solution of 0.5% fish gelatin, 0.5% Triton X-100 and 1% BSA. Samples were then incubated with primary antibodies diluted in the blocking solution. For control experiments, the investigated primary antibody was omitted while the following procedures were unchanged. Specific labeling was revealed with fluorescent secondary antibodies (1:200) in the blocking solution and combined with actin staining with Alexa-Fluor-594- or Alexa-Fluor-647-conjugated phalloidin (Fisher Scientific, Illkirch, France). Samples were observed with a laser scanning confocal microscope (LSM 5 LIVE DUO, Zeiss). Final image processing was performed with Adobe Photoshop software (San Jose, CA). Control reactions were observed and processed with the parameters used for the stained sections.

### Antibodies

Two different combinations of primary antibodies were used. Antibodies were used as cellular markers. In control ears, they revealed the expected cellular morphology and distribution, as demonstrated in previous publications. Table S2 provides details and the appropriate references. The first combination included the mouse monoclonal anti-synaptophysin antibody and the anti-calretinin goat serum. Synaptophysin is a protein expressed in small synaptic vesicles in efferent terminals, and at the top of calyx afferent terminals, and calretinin is a cytoplasmic Ca^2+^-binding protein expressed in most type II hair cells and pure calyx. In vestibular epithelia of adult rodents, calretinin specifically localizes at type II hair cells and some calyx nerve fibers and endings. This particular population of calretinin expressing calyces is restricted to the striolar zone of maculae and the central zone of cristae. This population is identified as the purely calyx one, which corresponds to only calyx units without bouton branching, like dimorphic afferent fibers (Desai et al., 2005a,b; Desmadryl and Dechesne, 1992). The other combination involved an anti-synapsin rabbit serum, recognizing the synapsin phosphoprotein expressed in synaptic vesicles of efferent terminals, and the mouse monoclonal anti-neurofilament-200-kDa, recognizing neuronal intermediate filaments of the cytoskeleton of afferent fibers. Fluorescent secondary antibodies used were donkey sera conjugated to Alexa-Fluor-488-, Alexa-Fluor-594- and Alexa-Fluor-647-conjugated anti-mouse, anti-rabbit and anti-goat IgG, respectively (1:700, Fisher Scientific).
